# The Comparison of the Adverse Events of Pentavalent Vaccine and DPT Vaccine in 2–6 Months Infants in Iran: A National Study

**DOI:** 10.5334/aogh.2449

**Published:** 2020-02-03

**Authors:** Zaher Khazaei, Ghobad Moradi, Seyed Mohsen Zahraei, Mohammad Mehdi Gouya, Elham Goodarzi, Fateme Yaghini, Daem Roshani

**Affiliations:** 1 Department of Public Health, School of Medicine, Dezful University of Medical Sciences, Dezful, IR; 2 Social Determinants of Health Research Center, Research Institute for Health Development, Kurdistan University of Medical Sciences, Sanandaj, IR; 3 Center for Communicable Diseases Control, Ministry of Health and Medical Education, Tehran, IR; 4 Social Determinants of Health Research Center, Lorestan University of Medical Sciences, Khorramabad, IR; 5 Department of Epidemiology and Biostatistics, Social Determinants of Health Research Center, Research Institute for Health Development, Kurdistan University of Medical Sciences, Sanandaj, IR

## Abstract

**Background:**

Vaccination is the most remarkable intervention in public health and is an effective strategy in controlling infectious diseases among infants.

**Objectives:**

The aim of this study was to compare the adverse events of Pentavalent vaccine and DPT vaccine in two- to six-month-old infants in Iran.

**Methods:**

This is an analytical cross-sectional study in which healthy infants aged two to six months, having received DPT vaccine in 2013 and Pentavalent vaccine in 2015, were studied for any experienced adverse events related to these vaccines. Percentage, mean, standard deviation and chi-square tests were used to describe and analyze the data (p < 0.05).

**Findings:**

The results showed that 10,464 and 17,561 adverse events, which were associated with DPT vaccine and Pentavalent vaccine respectively, were recorded in the infants who received these vaccines throughout Iran. Mazandaran, Qazvin and Golestan provinces reported the highest number of adverse events, respectively (15.74%, 11.25%, and 9.12%). Moreover, Pentavalent vaccine seemed to have more recorded adverse events compared to DPT, high fever had the highest record rate for DPT vaccine (47.4%) and mild localized complications was the highest for Pentavalent vaccines (31.68%). There was a significant relationship between the kind of vaccine and the type of reaction, adverse event categorization and the country that produced the vaccine (p < 0.05).

**Conclusion:**

Severe localized adverse events including high fever, vomiting, diarrhea and restlessness seemed to be less in Pentavalent vaccine compared to DPT vaccine. Therefore, substituting Pentavalent vaccine for DPT vaccine in infants seems to reduce the adverse events among them.

## Background

The World Health Organization (WHO) considers infant vaccination the most influential health intervention for promoting a healthy society [1]. Infant vaccination programs have been merged into public health service networks from their starting point in Iran. With 98% of infants vaccinated, it has brought great success in eradicating, removing and controlling preventable diseases [2,3,4]. Although modern and new vaccines used throughout the country are supposed to be safe, there is no vaccine without adverse effects. Each vaccine has its own side effects that might appear following their use [5]. Based on the WHO and Iran’s care guide recommendations, regardless of any causal relationship, every side effect observed in the vaccinated person by physicians, family members or the person himself is known as an adverse event following immunization [6]. Immunization adverse events can be due to errors in the vaccination program, reactions due to the nature of the vaccine, reactions to injection or unknown factors. Sometimes there might be some adverse events temporarily assigned to vaccination because of their concurrency [5,7].

Furthermore, the Pentavalent vaccine immunization program, which is used to prevent hepatitis B, Diphtheria, anthrax, Tetanus and Haemophilus influenzae type b (Hib) flu and is injected in three different time intervals (2, 4 and 6 months), started in October 2014 in Iran [8]. This vaccine is used broadly in more than 100 countries for preventing Diphtheria, Tetanus, and Pertussis (DPT), Hepatitis B and Hib in recent years. A study in the USA showed that fever (25.8%), injection site sensitivity (15.8%) and injection site edema (10.8%) were the most remarkable adverse events of Pentavalent vaccine [9,10]. Some of the Pentavalent vaccine advantages include reducing the number of injections and syringes used, less pain and restlessness in infants, decreasing the complications of injection, ease of planning, increasing cost effectiveness and increasing the immunization coverage [11].

However, no national study has been done on the possible adverse events of this vaccine in Iran since its merging into the country’s national vaccination program on November 22, 2014 [10]. This study attempted to broadly compare the possible adverse events of Pentavalent vaccine as a new vaccine in comparison to DPT vaccine in two- to six-month-old infants in Iran in 2016.

## Methods

This is an analytical cross-sectional study. All healthy infants (male and female of two to six months) who visited health centers to receive DPT vaccine from April 18 to March 25, 2013, as well as those who received Pentavalent vaccine from April 30 to March 25, 2015, and experienced adverse events following vaccination were studied. Those infants with allergic reactions or convulsions prior to vaccination and those who received immunosuppressive and neurological disorder medications were excluded from the study. Pentavalent vaccine and DPT vaccine used in this study had been produced in Iran, Korea, India, Indonesia, France and Belgium, and they are used broadly in the world. To perform vaccination, 0.5 mL was injected into the anterior part of the quadriceps muscle of the infants.

The recorded information includes the name of the province and medical university in the area, the date of the report, the city, the patient’s sex, the type of report (urgent vs. non-urgent), hospitalization status (outpatient vs. inpatient), type of reporting health center (urban vs. rural), infant’s age, their birth weight, birth date, immunization date, type of reaction to vaccination, the vaccine name, the vaccine serial number, the date of receiving the vaccine, the name of the institute or factory producing the vaccine, the adverse event incidence date, the patient’s visit date, their mother’s age in the time of pregnancy, the treatment procedure (recovery, being treated, lasting adverse event, death, other), the final diagnosis and the adverse event categorization (vaccine reaction, error in the vaccination program, simultaneous injection response, unknown). Because the infant’s recording information form was used to gather the required data and the data analysis was done in groups, there was not any ethical problem in this study; moreover, the researchers considered themselves to be committed to research ethics.

## Data Analysis

STATA version 12 was used to analyze the data. Percentage, mean, standard deviation and chi-square tests were used to describe the data and to investigate the studied variables. Significance level was considered to be p < 0.05.

## Findings

The results showed 10,464 adverse events (about 0.3%) among 4,249,050 infants who received DPT vaccine in 2013 and 17,561 adverse events (about 0.3%) among 423,0870 infants who received Pentavalent vaccine in 2015. The results also showed that 53.36% of the infants who experienced DPT vaccine adverse events were male and about 61.53% lived in rural areas (Table 1). In addition, the results showed that the average birth weight of these infants were 3160 ± 487 g and 3202 ± 455.2 g and their average gestational age were 38.4 ± 1.51 and 38.5 ± 1.31 weeks for DPT vaccine and Pentavalent vaccine, respectively.

**Table 1:** Demographic variables of infants with vaccine complications.

Variable		Vaccine	

		DPT	Pentavalent

Sex	Male	5584 (53.36)	**9137 (52.1)**
	Female	4880 (46.64)	**8399 (47.9)**
Location	Urban	4025 (38.47)	**8788 (50.04)**
	Rural	64399 (61.53)	**8773 (49.96)**

Moreover, the results showed that Mazandaran (15.74%), Ghazvin (11.25%), Golestan (9.12%) and Zanjan (8.44%) have reported the highest and Chaharmahal and Bakhtiari (0.08%), Hormozgan and Bushehr (0.21%), Kurdistan and South Khorasan (0.023%) have reported the fewest number of vaccination adverse events, respectively. The highest number of DPT adverse events were reported from Mazandaran (17.27%), Golestan (13.47%) and Qazvin (11.55%), and the fewest were reported from Chaharmahal and Bakhtiari (0.03%), South Khorasan (0.11%), Bushehr and Kurdistan (0.16%), respectively. Moreover, the highest number of Pentavalent vaccine adverse events were reported from Mazandaran (14.83%), Qazvin (11.05%) and Zanjan (8.01%), and the fewest were reported from Chaharmahal and Bakhtiari (0.11%), Hormozgan (0.22%), and Bushehr and Kurdistan (0.27%), respectively. This difference was not statistically significant (p > 0.05) (Table 2).

**Table 2:** The frequency of adverse events DPT vaccine and Pentavalent vaccine in different provinces of Iran.

ID	Province	Frequency of vaccination complications (%)		Total

		DPT	Pentavalent	

**1**	Mazandaran	1807 (17.27)	2605 (14.83)	4412 (15.74)
**2**	Qazvin	1211 (11.57)	1941 (11.05)	3152 (11.25)
**3**	Golestan	1409 (13.47)	1146 (6.53)	2555 (9.12)
**4**	Zanjan	958 (9.16)	1407 (8.01)	2365 (8.44)
**5**	Khuzestan	1019 (9.74)	1183 (6.74)	2202 (7.86)
**6**	Gilan	84 (8.04)	838 (4.77)	1676 (5.99)
**7**	Fars	469 (4.48)	994 (5.66)	1463 (5.22)
**8**	Isfahan	580 (5.54)	877 (4.99)	1457 (5.2)
**9**	Alborz	144 (1.38)	1202 (6.84)	1346 (4.8)
**10**	East Azerbaijan	290 (2.77)	819 (4.66)	1109 (3.96)
**11**	Khorasan Razavi	331 (3.16)	756 (4.3)	1087 (3.88)
**12**	Tehran	255 (2.44)	682 (3.88)	937 (3.34)
**13**	Sistan & Baluchestan	162 (1.55)	423 (2.41)	585 (2.09)
**14**	Lorestan	99 (0.95)	401 (2.28)	500 (1.78)
**15**	West Azerbaijan	162 (1.55)	250 (1.42)	412 (1.47)
**16**	Ardebil	17 (0.16)	379 (2.16)	396 (1.41)
**17**	Ilam	161 (1.54)	221 (1.26)	382 (1.36)
**18**	Yazd	102 (0.97)	193 (1.1)	295 (1.05)
**19**	Hamedan	93 (0.89)	179 (1.02)	272 (0.97)
**20**	Kermanshah	48 (0.46)	157 (0.89)	205 (0.73)
**21**	North Khorasan	39 (0.37)	143 (0.81)	182 (0.65)
**22**	Semnan	46 (0.44)	136 (0.77)	182 (0.65)
**23**	Kerman	53 (0.51)	126 (0.72)	179 (0.64)
**24**	Markazi	44 (0.42)	121 (0.69)	165 (0.59)
**25**	Qom	41 (0.39)	83 (0.47)	124 (0.44)
**26**	Kohgiluyeh & Boyer-Ahmad	13 (0.12)	95 (0.54)	108 (0.39)
**27**	Bushehr	17 (0.16)	47 (0.27)	64 (0.23)
**28**	Southern Khorasan	12 (0.11)	53 (0.3)	64 (0.23)
**29**	Kurdistan	17 (0.16)	47 (0.27)	64 (0.23)
**30**	Hormozgan	21 (0.2)	38 (0.22)	59 (0.21)
**31**	Chaharmahal & Bakhtiari	13 (0.12)	95 (0.54)	108 (0.39)
**32**	Total country	10464 (100)	1756 (100)	28025 (100)

**Figure 1:**
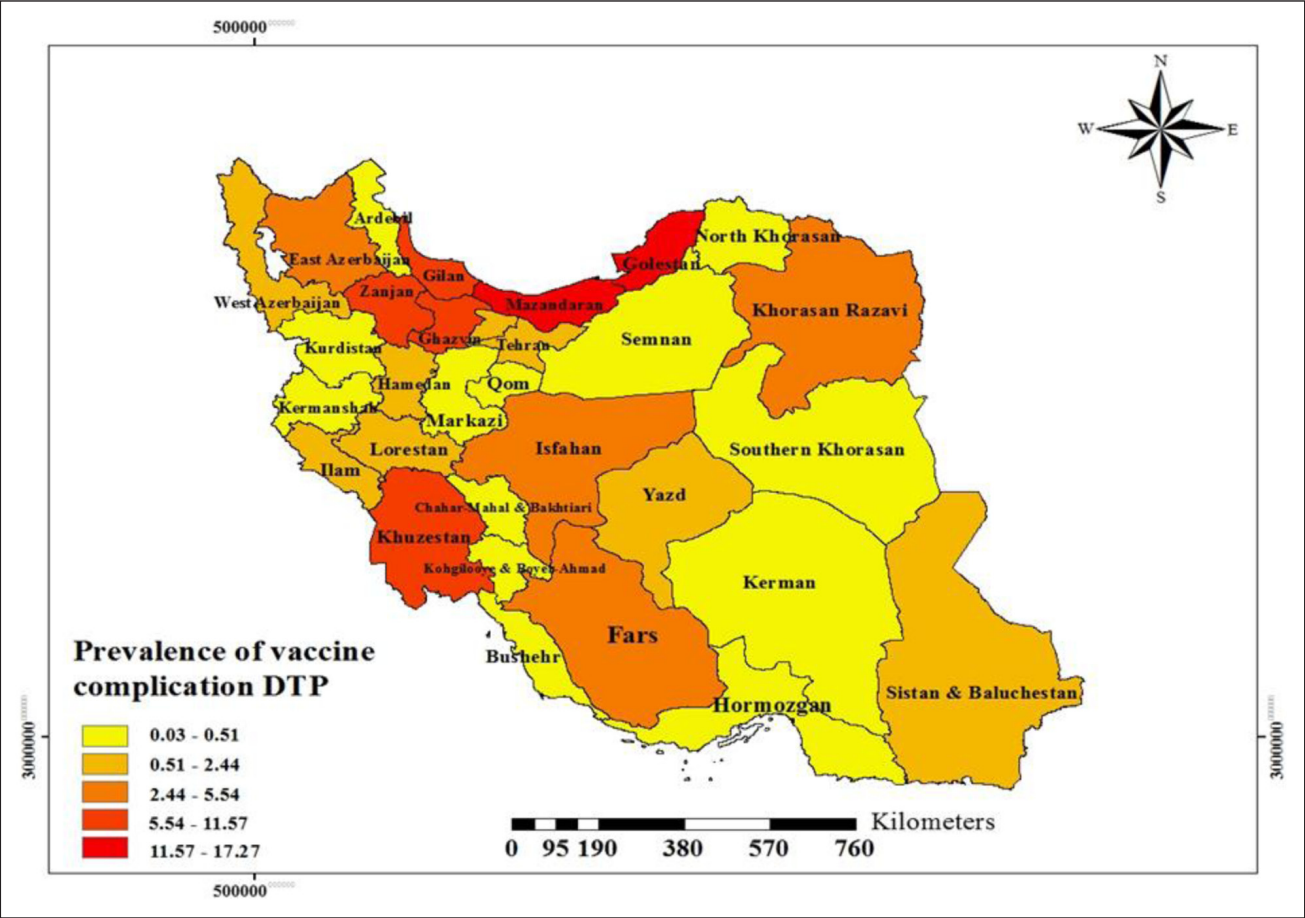
Prevalence of Adverse Events Following Immunization with DPT Vaccine.

**Figure 2:**
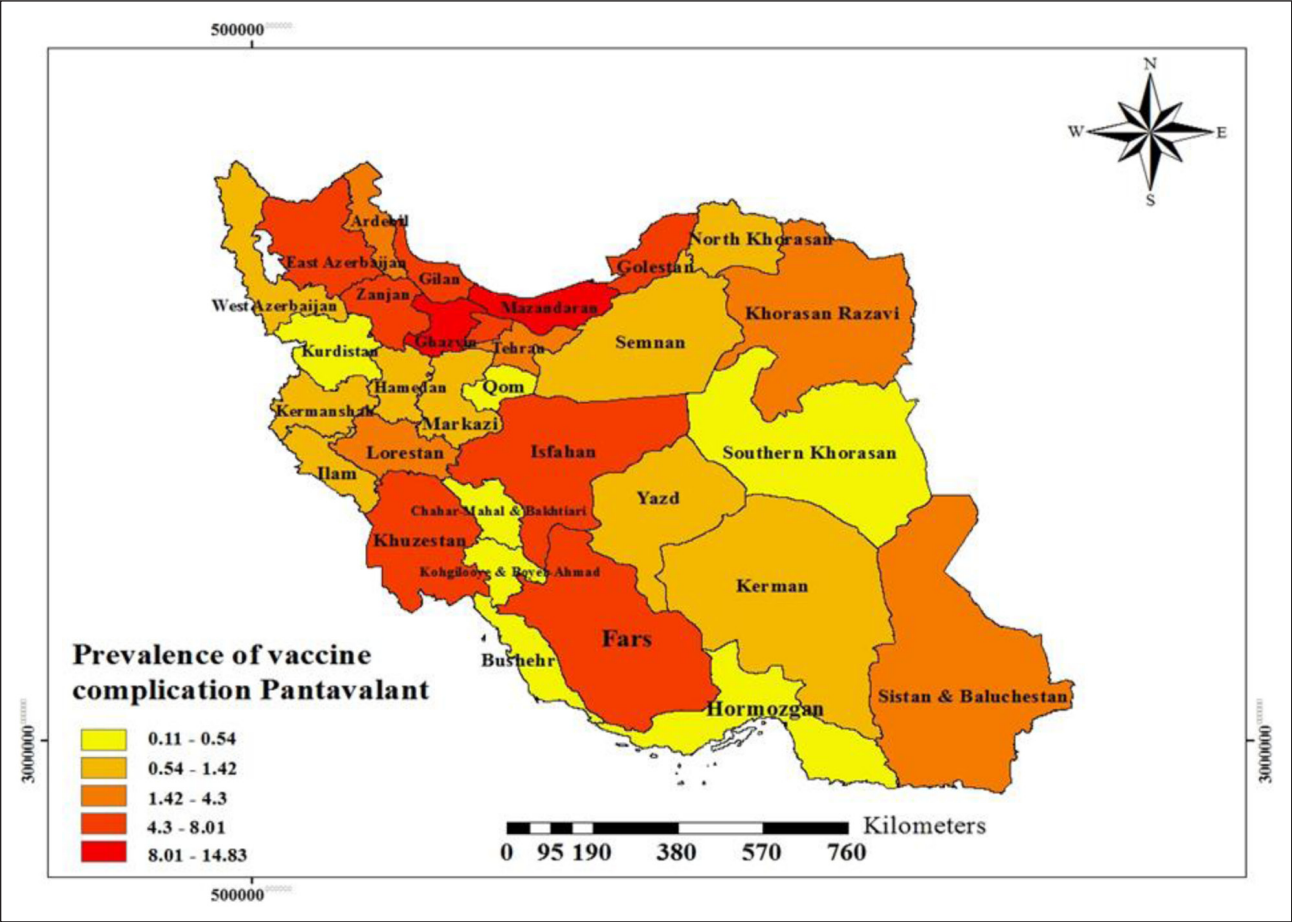
Prevalence of Adverse Events Following Immunization with Pentavalent Vaccine.

The dispersion of the DPT vaccine and Pentavalent vaccine adverse events in different parts of the country is shown using geographical information system in Figures 1 and 2. Most of the complications were in the northern provinces of the country.

**Figure 3:**
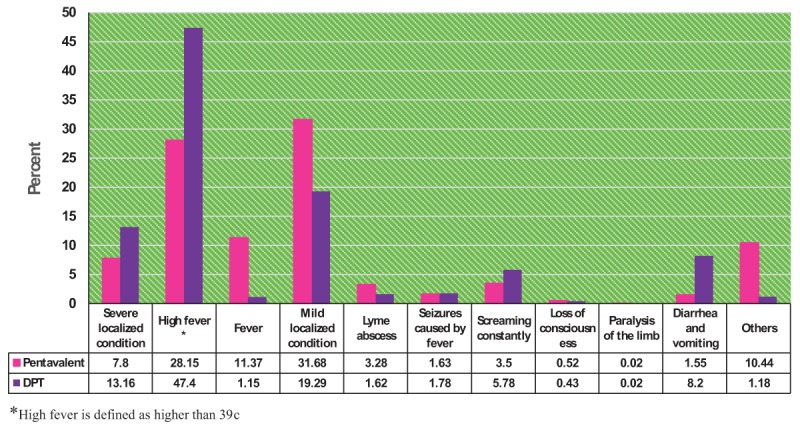
Comparison of Reported Adverse Events in DPT and Pentavalent Vaccines.

The results also showed that high fever (47.4%) and mild localized complications (31.68%) were reported to be the most frequent events for DPT vaccine and Pentavalent vaccine, respectively. Therefore, although more adverse events were reported for Pentavalent vaccine (17,561 vs. 10,464 cases) compared to DPT vaccine, most of the adverse events reported with Pentavalent vaccine were mild; whereas, adverse events reported with DPT vaccine were mostly high fever (p < 0.05) (Figure 3).

According to the results, only 3% of the adverse events in both vaccines led to hospitalization. Vaccine adverse event categorization in both DPT vaccine and Pentavalent vaccine showed that the largest category belonged to vaccine reaction (about 95%). The results also indicated that there was a statistically significant relationship between the kind of vaccine and the type of reaction, adverse event categorization and the infant’s place of residence (p < 0.05). However, there was no statistically significant relationship between the kind of vaccine and the infant’s sex or their hospitalization status (p < 0.05) (Table 3).

**Table 3:** The relationship between DPT and Pentavalent vaccine characteristics and their adverse events.

Variable		Type Vaccine		Chi-2	P-value

		DPT	Pentavalent		

Type of reaction	Severe localized condition	1375 (7.83)	1377 (13.16)	3.8	0.0001
	High fever*	4944 (28.15)	4960 (47.4)		
	Fever	1997 (11.37)	120 (1.15)		
	Mild localized condition	5564 (31.68)	2018 (19.29)		
	Lyme abscess	576 (3.28)	170 (1.62)		
	Seizures caused by fever	287 (1.63)	186 (1.78)		
	Screaming constantly	614 (3.5)	605 (5.78)		
	Loss of consciousness	91 (0.52)	45 (0.43)		
	Paralysis of the limb	3 (0.02)	2 (0.02)		
	Diarrhea and vomiting	277 (1.58)	858 (8.2)		
	Others	1833 (10.44)	123 (1.18)		
Complaint classification	Programming error	718 (4.4)	298 (2.85)	263.1	0.0001
	Reactions to vaccines	15099 (92.47)	10110 (96.63)		
	Reaction to the injection	287 (1.76)	489(0.46)		
	Concurrency	139 (0.85)	4 (0.04)		
	Unknown	85 (0.52)	3 (0.03)		
Hospitalization	Yes	157 (1.5)	420 (2.39)	0.11	0.7
	No	10307 (98.5)	17141 (61.97)		
Report type	Immediate	472 (4.51)	966 (5.5)	13.19	0.0001
	Non-Immediate	9992 (95.49)	16595 (94.5)		
Gender	Male	5584 (53.36)	9137 (52.1)	4.2	0.05
	Female	4880 (46.64)	8399 (52.1)		
Location	Urban	4025 (38.47)	8788 (47.9)	354	0.0001
	Rural	6439 (61.53)	8773 (49.96)		

* High fever is defined as higher than 39c.

## Discussion

Although vaccines used in the country’s immunization program are safe and very effective, no vaccine is completely safe and there might be some adverse events following immunization [12]. On the whole, the number of adverse events following immunization are low in Iran, and most are reported to be mild and temporary and mostly resolved without any medical treatment [13]. The results showed that the general incidence of adverse events in DPT vaccine and Pentavalent vaccine were 0.2% and 0.3%, respectively (p > 0.05). Among other adverse events, high fever and mild localized complication were reported to have the highest incidence in DPT vaccine (47.7%) and Pentavalent vaccines (31.68%).

The increase in adverse events reported with the Pentavalent vaccine in 2015 compared to adverse events reported with the DPT vaccine in 2013 could be explained by the general increase in the number of adverse events reported throughout the country and by the increased ability of the care and health system in identifying and recording more vaccine adverse events in 2015 compared to 2013.

In fact, at least one out of four infants having received the vaccines showed some kind of adverse event, which were mostly associated with fever [14]. Fever could generally be produced after receiving all kinds of vaccines. This study showed that high fever was the most common adverse event of DPT vaccine (47.4%); whereas, it was 28.15% for the Pentavalent vaccine.

Similarly, in studies by Sharifi et al. [9] and Ayatollahi et al. [15], high fever was reported to be the most frequent adverse event of the DPT vaccine. Mansour et al. in New Zealand reported longtime crying among infants who had received the DPT vaccine [16]. Al-Jadiryinin Iraq reported pain and inflammation as the most common events associated with the DPT vaccine [17]. Barkin et al. reported that 54% of infants who received the vaccine showed fever as the most common event [18]. In a study by Suser et al., localized pain, redness, fever and edema were reported to be the most common adverse events of DPT vaccine [19]. The benefits of childhood vaccines far outweigh any potential risks. Significant global data on vaccination showed that lack of Hib vaccination caused a significant amount of disease and mortality among infants. Therefore, WHO strongly recommends global Hib Vaccination [20]. Vaccination with the Pentavalent vaccine automatically increased the coverage of Hepatitis B and Hib immunization [11].

In addition, the results of different studies show that the Pentavalent vaccine was safe and tolerable and possessed a high level of immunizing for all molecular antigens and some biological reactions [21,22,23,24]. This vaccine was tested for 10 years in some Asian countries, from 2002 (when its use started in Ghana) to 2012 (when its use started in India) [24]. The results of these studies show that the most common reported adverse event of the Pentavalent vaccine was localized mild events. A study by the Indian Institute of Serology shows that local reactions to the Pentavalent vaccine included pain, redness and edema in the injection site, and its general systematic reactions were reported to be fever, unusual crying and irritability [25]. Hatami et al. in Tehran reported fever as the most common adverse event of the Pentavalent vaccine (71.2%) [13]. In a study in the United States of America, high fever was reported to be the most common adverse event of the Pentavalent vaccine [26]; whereas, Cunha et al. reported hypo-tony as the most common adverse event for the DPT vaccine and the Pentavalent vaccine [27].

Finally, it should be noted that most parents were aware of the fever following vaccination and they preventively gave Acetaminophen to their infants, which could decrease their fever and prevent high fever; therefore, the incidence rate of this event might be changed due to the parents’ interventions [14]. Categorizing the adverse events showed that most of the reported events were associated with the reaction to the vaccine, which was in line with Raisi et al. in Shahre Kurd [28] and Parisay et al. in Kohkiloye va Boir Ahmad [29]. In addition, the findings of the study showed that there was not any significant relationship between the infants’ genders and the DPT vaccine and Pentavalent vaccine adverse events. However, the possible complications of the vaccines seemed to be more common among males compared to females. This finding was in line with the findings of Reisi et al. in which there wasn’t a significant relationship between the gender of the infants and the vaccines’ adverse events [28]. Moreover, in studies by Parisay et al. [29] and Ayatollahi et al. [30], although there was not a significant relationship between the infants’ genders and the vaccines’ adverse events, the possible adverse events were reported to be more common among males compared to females, which was in line with the findings of this study. However, Nabavi et al. reported that the vaccines’ adverse events seemed to be more common among females than males, which is in contradiction to the findings of this study [31].

However, severe cases of adverse events led to hospitalization for a small percentage of the vaccinated infants. The findings of the study show that only 1.5% of all reported complications in infants receiving the DPT vaccine and the Pentavalent vaccine led to their hospitalization and the number hospitalized were almost the same for both vaccines. In fact, many hospitalizations can be due to other factors contemporarily existing with vaccination.

## Conclusion

In conclusion, the Pentavalent vaccine demonstrated fewer adverse events and reduced the number of injections and the required injection equipment, showing that this vaccine is better than the DTP vaccine.
